# Reidentification of Ebola Virus E718 and ME as Ebola Virus/H.sapiens-tc/COD/1976/Yambuku-Ecran

**DOI:** 10.1128/genomeA.01178-14

**Published:** 2014-11-20

**Authors:** Jens H. Kuhn, Loreen L. Lofts, Jeffrey R. Kugelman, Sophie J. Smither, Mark S. Lever, Guido van der Groen, Karl M. Johnson, Sheli R. Radoshitzky, Sina Bavari, Peter B. Jahrling, Jonathan S. Towner, Stuart T. Nichol, Gustavo Palacios

**Affiliations:** a Integrated Research Facility at Fort Detrick (IRF-Frederick), National Institute of Allergy and Infectious Diseases, National Institutes of Health, Fort Detrick, Frederick, Maryland, USA; b United States Army Medical Research Institute of Infectious Diseases (USAMRIID), Fort Detrick, Frederick, Maryland, USA; c Biomedical Sciences Department, Dstl, Porton Down, Salisbury, Wiltshire, United Kingdom; d Prins Leopold Instituut voor Tropische Geneeskunde (ITG), Antwerp, Belgium; e Retired, Portland, Oregon, USA; f Viral Special Pathogens Branch, Division of High-Consequence Pathogens Pathology, National Center for Emerging and Zoonotic Infectious Diseases, Centers for Disease Control and Prevention (CDC), Atlanta, Georgia, USA

## Abstract

Ebola virus (EBOV) was discovered in 1976 around Yambuku, Zaire. A lack of nomenclature standards resulted in a variety of designations for each isolate, leading to confusion in the literature and databases. We sequenced the genome of isolate E718/ME/Ecran and unified the various designations under Ebola virus/H.sapiens-tc/COD/1976/Yambuku-Ecran.

## GENOME ANNOUNCEMENT

Ebola virus (EBOV) was discovered in 1976 around Yambuku, Équateur Province, Zaire (the present-day Democratic Republic of the Congo). Of the 318 people that became infected with the virus, 280 died (1). At least three EBOV isolates (Ecran, de Roover, and Mayinga) were obtained and distributed among laboratories for further characterization. Due to a lack of nomenclature standards, individual institutes assigned a variety of names to each individual isolate, thereby leading to confusion in the literature and in databases.

Filovirus experts recently established a naming scheme for filoviruses (2, 3) and have designated reference isolates and sequences for each filovirus to facilitate research, automatic genome annotation, and database searches (4). Among these efforts are attempts to identify and remove possibly redundant isolate designations.

Isolate E718 (known as 718 [5], E718 [6–14), E-718 [15–17], and E 718 [6, 15, 18–21]) is one of the EBOV isolates found in 1976. The origin of E718 is usually not mentioned, or it is stated only that E718 was derived from an acute-phase human blood sample (5, 10–14, 16, 17, 20, 21). Other authors elaborate that the isolate was obtained by S. R. Pattyn (Instituut voor Tropische Geneeskunde [ITG], Antwerp, Belgium) (6, 15, 18). One article refers to the isolate as the Zaire prototype (8), whereas a very recent publication states that “E718 [is] … one of four isolates (including … Mayinga)” (9). Finally, one publication states that E718 “was derived from a Zairean patient (ME) during the Zairean outbreak” (7). One of two publications detailing isolate ME states that this virus was “isolated by… Pattyn … Bowen, and … Webb from serum of adult female human being manifesting severe … illness” and that “[d]isease [was] contracted in Yambuku … and case transported to mission hospital in Kinshasa” (22). Other publications specify that patient ME was a 42-year-old woman who fell ill on 23 September 1976 in Yambuku and was transported by air to Kinshasa (23, 24). She died on 30 September 1976 (25, 26).

From a review of internal documents at institutes that still store E718 cultures (CDC, Defence Science and Technology Laboratory [Dstl], ITG, and the United States Army Medical Research Institute of Infectious Diseases [USAMRIID]), detailed descriptions of the 1976 outbreak (25, 26), and personal observations of authors who participated in its control (K.M.J. and G.v.d.G.), we conclude that E718 is a synonym of ME. Both designations are, in all likelihood, synonyms of the EBOV variant designation Ecran (often incorrectly spelled Eckron in the literature), which is derived from the name of Sister Myriam (Louise Ecran), who was one of two nurses that were transported from Yambuku to Kinshasa (the other one being Sister Edmonda [Jeanne de Roover]) (25, 26).

We sequenced E718 using infected cell-culture supernatant originally frozen at USAMRIID on 19 May 1978. Following sample preparation performed as described in Kugelman et al. (27), its sequence was determined with Ion Torrent PGM and Applied Biosystems Sanger technology with EBOV-specific oligonucleotides. The consensus genome was generated via reference alignment to that of RefSeq accession no. NC_002549 (Ebola virus/H.sapiens-tc/COD/1976/Yambuku-Mayinga [4]) using SeqMan Pro (DNAStar). As expected, the obtained sequence was nearly identical with that of RefSeq accession no. NC_002549 and has been deposited under the unifying designation Ebola virus/H.sapiens-tc/COD/1976/Yambuku-Ecran.

### Nucleotide sequence accession numbers.

The GenBank accession no. of EBOV E718, now designated Ebola virus/H.sapiens-tc/COD/1976/Yambuku-Ecran, is KM655246.

## References

[1] World Health Organization 1978 Ebola haemorrhagic fever in Zaire, 1976. Bull. World Health Organ. 56:271–293. 307456 PMC2395567

[2] Kuhn JH Becker S Ebihara H Geisbert TW Johnson KM Kawaoka Y Lipkin WI Negredo AI Netesov SV Nichol ST Palacios G Peters CJ Tenorio A Volchkov VE Jahrling PB 2010 Proposal for a revised taxonomy of the family *Filoviridae*: classification, names of taxa and viruses, and virus abbreviations. Arch. Virol. 155:2083–2103. 10.1007/s00705-010-0814-x. 21046175 PMC3074192

[3] Kuhn JH Bao Y Bavari S Becker S Bradfute S Brister JR Bukreyev AA Chandran K Davey RA Dolnik O Dye JM Enterlein S Hensley LE Honko AN Jahrling PB Johnson KM Kobinger G Leroy EM Lever MS Mühlberger E Netesov SV Olinger GG Palacios G Patterson JL Paweska JT Pitt L Radoshitzky SR Saphire EO Smither SJ Swanepoel R Towner JS van der Groen G Volchkov VE Wahl-Jensen V Warren TK Weidmann M Nichol ST 2013 Virus nomenclature below the species level: a standardized nomenclature for natural variants of viruses assigned to the family *Filoviridae*. Arch. Virol. 158:301–311. 10.1007/s00705-012-1454-0. 23001720 PMC3535543

[4] Kuhn JH Andersen KG Bào Y Bavari S Becker S Bennett RS Bergman NH Blinkova O Bradfute S Brister JR Bukreyev A Chandran K Chepurnov AA Davey RA Dietzgen RG Doggett NA Dolnik O Dye JM Enterlein S Fenimore PW Formenty P Freiberg AN Garry RF Garza NL Gire SK Gonzalez JP Griffiths A Happi CT Hensley LE Herbert AS Hevey MC Hoenen T Honko AN Ignatyev GM Jahrling PB Johnson JC Johnson KM Kindrachuk J Klenk HD Kobinger G Kochel TJ Lackemeyer MG Lackner DF Leroy EM Lever MS Mühlberger E Netesov SV Olinger GG Omilabu SA Palacios G 2014 Filovirus RefSeq entries: evaluation and selection of filovirus type variants, type sequences, and Names. Viruses 6:3663–3682. 10.3390/v6093663. 25256396 PMC4189044

[5] van der Groen G Jacob W Pattyn SR 1979 Ebola virus virulence for newborn mice. J. Med. Virol. 4:239–240. 10.1002/jmv.1890040309. 536744

[6] Bowen ETW Lloyd G Platt G McArdell LB Webb PA Simpson DIH 1978 Virological studies on a case of Ebola virus infection in man and in monkeys, p 95–102 *In* Pattyn SR (ed), Ebola virus haemorrhagic fever: proceedings of an international colloquium on Ebola virus infection and other haemorrhagic fevers held in Antwerp, Belgium, 6–8 December, 1977. Elsevier/North-Holland Biomedical Press, Amsterdam, the Netherlands.

[7] Ellis DS Stamford S Lloyd G Bowen ET Platt GS Way H Simpson DI 1979 Ebola and Marburg viruses: I. Some ultrastructural differences between strains when grown in Vero cells. J. Med. Virol. 4:201–211. 10.1002/jmv.1890040306. 94087

[8] Bowen ET Platt GS Lloyd G Raymond RT Simpson DI 1980 A comparative study of strains of Ebola virus isolated from southern Sudan and northern Zaire in 1976. J. Med. Virol. 6:129–138. 10.1002/jmv.1890060205. 6165800

[9] Piercy TJ Smither SJ Steward JA Eastaugh L Lever MS 2010 The survival of filoviruses in liquids, on solid substrates and in a dynamic aerosol. J. Appl. Microbiol. 109:1531–1539. 10.1111/j.1365-2672.2010.04778.x. 20553340

[10] Smither SJ Piercy TJ Eastaugh L Steward JA Lever MS 2011 An alternative method of measuring aerosol survival using spiders’ webs and its use for the filoviruses. J. Virol. Methods 177:123–127. 10.1016/j.jviromet.2011.06.021. 21762730

[11] Lever MS Piercy TJ Steward JA Eastaugh L Smither SJ Taylor C Salguero FJ Phillpotts RJ 2012 Lethality and pathogenesis of airborne infection with filoviruses in A129 α/β -/- interferon receptor-deficient mice. J. Med. Microbiol. 61:8–15. 10.1099/jmm.0.036210-0. 21852521

[12] Smither SJ Eastaugh LS Steward JA Nelson M Lenk RP Lever MS 2014 Post-exposure efficacy of oral T-705 (favipiravir) against inhalational Ebola virus infection in a mouse model. Antiviral Res. 104:153–155. 10.1016/j.antiviral.2014.01.012. 24462697

[13] Fisher-Hoch SP Platt GS Lloyd G Simpson DI Neild GH Barrett AJ 1983 Haematological and biochemical monitoring of Ebola infection in rhesus monkeys: implications for patient management. Lancet 2:1055–1058. 6138602 10.1016/s0140-6736(83)91041-3

[14] Fisher-Hoch SP Platt GS Neild GH Southee T Baskerville A Raymond RT Lloyd G Simpson DI 1985 Pathophysiology of shock and hemorrhage in a fulminating viral infection (Ebola). J. Infect. Dis. 152:887–894. 10.1093/infdis/152.5.887. 4045253

[15] Ellis DS Bowen ET Simpson DI Stamford S 1978 Ebola virus: a comparison, at ultrastructural level, of the behaviour of the Sudan and Zaire strains in monkeys. Br. J. Exp. Pathol. 59:584–593. 106868 PMC2041404

[16] Moe JB Lambert RD Lupton HW 1981 Plaque assay for Ebola virus. J. Clin. Microbiol. 13:791–793. 7014628 10.1128/jcm.13.4.791-793.1981 PMC273881

[17] Lupton HW Lambert RD Bumgardner DL Moe JB Eddy GA 1980 Inactivated vaccine for Ebola virus efficacious in guineapig model. Lancet 2:1294–1295. 6108462 10.1016/s0140-6736(80)92352-1

[18] Bowen ET Platt GS Simpson DI McArdell LB Raymond RT 1978 Ebola haemorrhagic fever: experimental infection of monkeys. Trans. R. Soc. Trop. Med. Hyg. 72:188–191. 10.1016/0035-9203(78)90058-5. 418537

[19] Bowen ETW Baskerville A Cantell K Mann GF Simpson DIH Zuckerman AJ 1978 The effect of interferon on experimental Ebola virus infection in rhesus monkeys, p 245–253 *In* Pattyn SR (ed), Ebola virus haemorrhagic fever: proceedings of an international colloquium on Ebola virus infection and other haemorrhagic fevers held in Antwerp, Belgium, 6–8 December, 1977. Elsevier/North-Holland Biomedical Press, Amsterdam, the Netherlands.

[20] Lupton HW 1981 Inactivation of Ebola virus with ^60^Co irradiation. J. Infect. Dis. 143:291. 10.1093/infdis/143.2.291. 7217722

[21] Baskerville A Fisher-Hoch SP Neild GH Dowsett AB 1985 Ultrastructural pathology of experimental Ebola haemorrhagic fever virus infection. J. Pathol. 147:199–209. 10.1002/path.1711470308. 4067737

[22] Pattyn SR 1978 Ebola (EBO): strain: ME. Am. J. Trop. Med. Hyg. 27:383–384.

[23] Pattyn S van der Groen G Courteille G Jacob W Piot P 1977 Isolation of Marburg-like virus from a case of haemorrhagic fever in Zaire. Lancet 1:573–574. 65663 10.1016/s0140-6736(77)92002-5

[24] Pattyn SR Bowen ETW Webb PA 1985 Ebola, p 379–380 *In* Karabatsos N (ed), International Catalogue of Arboviruses 1985 including certain other viruses of Vertebrates, 3rd ed. American Society of Tropical Medicine and Hygiene, San Antonio, TX.

[25] Garrett L 1994 Yambuku—Ebola, p 100–152 *In* Garrett L (ed), The coming plague: newly emerging diseases in a world out of balance. Farrar, Straus & Giroux, New York, NY.

[26] Sureau P 1977 Yambuku. A novel African hemorrhagic fever. The discovery of the Ebola virus. The Yambuku epidemic, Zaire, September–October 1976. Travel log book. Paris, France.

[27] Kugelman JR Lee MS Rossi CA McCarthy SE Radoshitzky SR Dye JM Hensley LE Honko A Kuhn JH Jahrling PB Warren TK Whitehouse CA Bavari S Palacios G 2012 Ebola virus genome plasticity as a marker of its passaging history: a comparison of *in vitro* passaging to non-human primate infection. PLoS One 7:e50316. 10.1371/journal.pone.0050316. 23209706 PMC3509072

